# Three Biopolymers and Origin of Life Scenarios

**DOI:** 10.3390/life14020277

**Published:** 2024-02-18

**Authors:** Ilana Agmon

**Affiliations:** 1 Institute for Advanced Studies in Theoretical Chemistry, Schulich Faculty of Chemistry, Technion—Israel Institute of Technology, Haifa 3200003, Israel; chilana@technion.ac.il; 2 Fritz Haber Research Center for Molecular Dynamics, Hebrew University, Jerusalem 9190401, Israel

**Keywords:** aminoacylation, genetic code, origin of life, proto-ribosome, ribosome evolution, RNA world, translation, tRNA

## Abstract

To track down the possible roots of life, various models for the initial living system composed of different combinations of the three extant biopolymers, RNA, DNA, and proteins, are presented. The suitability of each molecular set is assessed according to its ability to emerge autonomously, sustain, and evolve continuously towards life as we know it. The analysis incorporates current biological knowledge gained from high-resolution structural data and large sequence datasets, together with experimental results concerned with RNA replication and with the activity demonstrated by standalone constructs of the ribosomal Peptidyl Transferase Center region. The scrutiny excludes the DNA–protein combination and assigns negligible likelihood to the existence of an RNA–DNA world, as well as to an RNA world that contained a replicase made of RNA. It points to the precedence of an RNA–protein system, whose model of emergence suggests specific processes whereby a coded proto-ribosome ribozyme, specifically aminoacylated proto-tRNAs and a proto-polymerase enzyme, could have autonomously emerged, cross-catalyzing the formation of each other. This molecular set constitutes a feasible starting point for a continuous evolutionary path, proceeding via natural processes from the inanimate matter towards life as we know it.

## 1. Introduction

A fundamental argument opposing the autonomous advent of life via natural processes stresses the improbability that the complex life-components could have emerged randomly. This claim can be contested by presenting a significantly simpler version of life as we know it, which could have executed the key processes in a considerably less efficient and accurate manner than if carried out in modern life. Such a model, which combines relatively simple molecules into a self-sustained system, is required to emerge autonomously and to evolve into the extremely complex extant life.

The hub of contemporary life is the translation system, which is the best-preserved relic of the last common universal ancestor (LUCA) [1,2,3]. Comparative genomic studies revealed that essential genes, i.e., those common to all three domains of life, have by far the largest number of genes associated with translation [4,5,6]. In accordance, over half of the coding genes in the smallest living artificial cell yet generated, which contains less than 300 assigned genes, are affiliated with translation [7]. The capability to present a feasible scenario for the autonomous outset of a simple version of the extant translation system is therefore an indispensable condition for maintaining life emergence via natural processes.

The term “unit of evolution” was coined by John Maynard Smith [8] for a population of entities with the properties of multiplication, variation, and heredity, being thus bound to evolve and proliferate. This term will be used here for the initial, self-emerging molecular system, which is assumed to have evolved into the present life. It is taken as a premise here that the initial unit of evolution was encapsulated in an abiotic compartment of some sort ([9] and refs therein) which prompted the linkage among its components. Additionally, even the simplest unit of evolution is assumed to have included a catalytic component and genetic material encoding it, which, by going through amplification, variation, and selection, would have established a self-sustained system capable of undergoing Darwinian evolution.

Life as we know it can be classified in terms of macromolecular chemistry as an amino and nucleic acid system, which comprises three biopolymers: proteins, RNA, and DNA. Feasible nonenzymatic processes that yielded the monomers of all three polymer-types, under assumed prebiotic conditions, were demonstrated. Single amino acids, the building blocks of the proteins, were readily formed in the laboratory under simple conditions, using different sources of energy such as electric discharge, UV, shockwaves, X-rays, or heat ([10,11] and refs therein). Also, the occurrence of aromatic amino acids formed abiotically and subsequently preserved at depth beneath the Atlantis Massif was demonstrated [12], while the traces of eight standard amino acids were found in meteorites [13], exemplifying the formation of amino acids via natural processes. The spontaneous ligation of amino acids into polypeptides of up to 50 mer long was shown to occur on illite surfaces [14]. The building blocks of RNA and DNA, nucleotides and deoxynucleotides, have been synthesized in large amounts in a prebiotically plausible manner [15,16] and their ability to autonomously concatenate into random oligomers, in the 20–50 mer range, has been shown under natural conditions [14,15]. Moreover, ligation of short oligonucleotides into chains of about 200 mer was demonstrated theoretically to occur under a thermal gradient [17], while nucleotide chains of 100–300 mer were found to form on rock glasses [18].

Two main models for the advent of life, relying on the distinctive properties of the extant biopolymers composing them, were put forward. The first model, the RNA world hypothesis [19,20,21,22,23,24,25], was based on the idea that abiotically synthesized RNA strands could have acted as both the genetic material and the catalysts, to form a primordial unit of evolution. According to the second scenario, life started in an RNA–protein world where both components coevolved [3,26,27,28,29,30,31,32], cross-catalyzing the formation of each other, thus conforming to the principle of molecular mutualism [3].

Here, hypothetic units of evolution, composed of different combinations of the three biopolymers, are analyzed. Each combination is examined according to its feasibility to spontaneously emerge in the prebiotic era, to sustain, and to evolve towards life as we know it. The analysis incorporates insight gained from large sequence datasets and from high-resolution structural data, together with experimental results concerned with RNA replication and with the catalytic activity demonstrated by standalone analogs of the ribosomal core. The scrutiny assigns negligible likelihood to the existence of an extended RNA world, i.e., one that contained a replicase made of RNA. It excludes other biopolymer combinations, and points to the onset of an RNA–protein world as the feasible starting point for life as we know it.

## 2. Results

### 2.1. How Far Can the “Chemical Era” Go?

Before discussing possible scenarios for the emergence of life, it can be beneficial to examine what types of molecules could have autonomously materialized during the “chemical era”, prior to the advent of biological catalysts. It is taken as a premise here that the prebiotic macromolecules were composed of the same monomers as the modern ones. The spontaneous formation of all three types of extant biopolymers, under prebiotically plausible conditions, was already demonstrated [10,11,12,14,15,16,17,18]. The “chemical era” could have therefore exhibited a variety of oligonucleotides and polypeptides of random composition, where a fraction of them would fold into three-dimensional structures, achieving enhanced protection against degradation. Random polypeptides longer than 30 amino acids can fold into stable structures that perform enzymatic activity [33,34,35], while RNA chains as short as 20 mer can assume a compact form by base pairing, and apply catalysis [36,37]. Specifically, random RNA sequences of 25–40 residues were found by simulation to fold primarily into hairpins with a single or double stem-loop [38]. Consequently, a surrounding holding large amount of RNA oligonucleotides with random sequences and varied lengths would have contained a rich repertoire of self-emerging, weakly active RNA catalysts, together with transiently-appearing enzymes, which could have arbitrarily interacted with each other.

Directed evolution experiments demonstrated how RNA acquires resistance to cleavage by RNases in a test tube [39], establishing that Darwinian evolution can be carried out as a purely chemical process [40]. Analogously, enrichment of the portion of RNA sequences prone to form stable base-paired duplexes and tertiary structures could have spontaneously taken place in the prebiotic pool. Primordial cleaving elements such as Mg^2+^ ions in combination with a buffer [41], or autonomously-materializing cleaving parasites made of RNA, such as the RNA moiety of RNAase P [42], would have first degraded the single-stranded RNA chains, enriching the pool with spontaneously-folded RNA oligomers; hence, with transiently appearing RNA catalysts.

Only the existence of an efficient and accurate enough means of RNA replication could have secured the constant presence of a transient ribozyme in the unit of evolution. However, even in the absence of a reliable manner of replication, the chemistry era could have yielded, via the spontaneous formation of RNA hairpins and L-shaped RNA molecules, simple versions of key components of the current translation system, i.e., the proto-tRNAs and the L-shaped monomers proposed to assemble the ancestral Peptidyl Transferase Center (PTC) [29]. Additionally, positively charged random peptides could have neutralized the negative charge on the RNA backbone, stabilizing its tertiary structure. Elements from this rich selection of arbitrarily formed molecules could have later been recruited for functional tasks in the upcoming prebiotic “worlds”.

### 2.2. Prebiotic “Worlds”

To form a self-sustained chemical system capable of undergoing Darwinian evolution that will eventually lead towards life as we know it, any initial unit of evolution would be required to contain both a genetic component and a catalytic component. Each of the three biopolymers that could have composed this initial unit, i.e., proteins, RNA, and DNA, possesses different properties, which determine its suitability to function within the units of evolution.

Complementary nucleotides from two DNA or RNA chains can specifically recognize one another via Watson–Crick base pairing; thus, they can be accurately template-replicated. DNA is a better information carrier than RNA due to its greater stability against hydrolysis [43], its lower tendency for self-folding [44], and its higher fidelity in replication [45]. On the other hand, DNA’s catalytic abilities, although existing, are lower than RNA’s, largely due to its inaptitude to form non-Watson–Crick base pairs. Being formed solely via Watson–Crick base pairing hinders its folding into complex 3D structures, which are essential for placing substrates in the required stereochemistry. An RNA string can be reliably replicated, but with less fidelity than DNA [45]. Additionally, it possesses catalytic abilities [46], although with significantly lower efficiency than proteins. Proteins are highly diverse in their competence to form defined structures, in their functional versatility, and in their catalytic efficiency [47,48], but the capability of peptides to serve as template molecules for their own replication is limited to specific sequences and conformations [26].

To assess the sustainability of units of evolution composed of different combinations of the three biopolymers, each component within the set is qualitatively assigned with two asterisks for excellent performance in a specific role, with a single asterisk for moderate functioning and with zero asterisks for performance level, which is significantly lower relative to the other biopolymers. DNA is assigned with two asterisks as an information-keeper, but with zero asterisks as a catalyst. RNA is assigned with a single asterisk as an information-keeper and with a single asterisk as a catalyst. Proteins, i.e., folded polypeptides, are assigned no asterisk as information-keepers, but two asterisks as catalysts. The final sustainability rank is determined by the multiplication of the number of asterisks assigned to the genetic role and to the catalytic role (Table 1).

This crude ranking method suggests that the probability of survival of a system composed solely of proteins or solely of DNA is negligible, due to the lack of an indispensable component, i.e., a reliable manner for storing genetic information in a protein-only set, and efficient enough catalytic abilities in a DNA-only set. At the other extremity, the system combining all three biopolymer types, which is an abstraction of life as we know it, seems to be the most sustainable set under the current environmental conditions. Evidently, this complex system could not have materialized spontaneously in the prebiotic world. It should have been predated by a simpler system, whose four optional sets are listed in Table 1 (sets 3–6); i.e., RNA-only, RNA–DNA, RNA–protein, and DNA–protein. These molecular sets are analyzed below for their likelihood to emerge spontaneously and to evolve continuously towards life as we know it.

#### 2.2.1. RNA-Only

An RNA-only unit of evolution is composed of RNA, which acts as both the genetic and the catalytic component. This molecular set is the equivalent of the widely accepted idea of an “RNA world” [19,20,21,22,23,24,25]. According to Table 1 it has a viable probability of survival, but its odds are the lowest among the tested sets.

##### Unit of Evolution Content

Except for random RNA chains, the sole obligatory component in an RNA-only unit of evolution is a means of copying RNA sequences, thus generating more copies of the randomly-emerging catalysts, and of the RNA-genome, when established. To sustain, replication was required to occur with minimum fidelity defined by the “error threshold” [26] that would have prevented the corruption of the encoded genetic information. Additional ribozymes to a replicase were suggested to form in the RNA world, e.g., a ribozyme that catalyzed the synthesis of the RNA nucleotides [49], a ribozyme that converted activated nucleotides to an ensemble of random-sequence polynucleotides [50], and the ancestor of the RNA moiety of the modern RNAase P that cleaved RNA [42]. These ribozymes would have granted an evolutionary advantage, but none of them seem to be indispensable for the sustainability of the RNA unit of evolution.

##### Autonomous Advent

A unit of evolution made solely of RNA could have materialized spontaneously in a surrounding holding large amount of RNA strings with random sequences and varied lengths. The chemistry era could have already yielded transiently appearing ribozymes with diverse catalytic activities. However, imperative to the RNA world hypothesis is the premise that RNA sequences would have been replicated within the unit of evolution via two template-directed copying steps.

Two modes of prebiotic replication, enzymatic and nonenzymatic, were hypothesized in this context. The first mechanism, i.e., enzymatic copying by a spontaneously materializing replicase made of RNA, elicited wide-scale in vitro directed evolution experiments, aimed at generating such a ribozyme. Two noteworthy results yielded large ribozymes of about 200 nucleotides each. One of them could copy a limited number of RNA strings having lengths comparable to its own, though not its sequence or its complementary one [51]. The second one could have significantly amplified short RNA strings but copied complex functional RNAs with low yield [52]. An alternative, purely chemical nonenzymatic template replication of DNA and RNA strings was demonstrated under assumed prebiotic conditions. It involved primer extension by monomer addition, as well as one-pot replication by template-directed ligation of nucleotide triplets and quartets ([53] and refs therein). The nonenzymatic template replication, providing it was efficient enough, would have bypassed the necessity for a replicase made of RNA.

RNA duplexes produced during replication could be separated by alteration of environmental factors such as pH cycling, evaporation–wetting cycles, oscillation of salt concentrations, and elevated temperatures [54], but long RNA strands tend to reform immediately. Experimental results, however, demonstrated that replication performed in a viscous environment allows strand separation, particularly for long, structured sequences like those of ribozymes [55].

##### Evolutionary Prospect

The sequences of all ribozymes in the RNA-only unit of evolution would have been concatenated into an early RNA genome that could instruct the synthesis of any ribozyme via two template-directed copying. Additional ribozymes would join through the spontaneous folding of random RNA strings and would be incorporated into the initial genome. Inaccuracies accumulated due to the primitive replication mode would enrich the sequence diversity, enabling Darwinian evolution and selection of the fittest ribozymes within each compartment, while the emergence of a new functional ribozyme would have granted its compartment with an evolutionary advantage over the neighboring compartments.

Subsequently, a second biopolymer would be incorporated into the RNA-only unit of evolution. This could have possibly been DNA, whose addition could be implemented via template-directed copying of the RNA genome into DNA, thus granting the RNA-only unit with improved sustainability (Table 1, set 4). Aside from the possible addition of DNA, an indispensable evolutionary step would take place prior to the advent of LUCA, that is, the incorporation of functional peptides into the unit of evolution. This was suggested to occur by proteins taking over most of the catalysis performed by RNA [56], resulting in a more sustainable set (Table 1, set 5), i.e., the RNA–protein unit of evolution, where the translation system would first materialize.

##### Extant Perspective

Only a single ribozyme, the ribosome, which is composed of a large subunit (LSU) and a small subunit (SSU), plays a key role in the modern replication and translation processes. However, in vitro directed evolution yielded, apart from the RNA replicases, many functional ribozymes, including those performing translation system roles, such as amino acid activation, RNA aminoacylation, and peptidyl transfer ([28] and refs therein), suggesting that similar ribozymes could have been active in the prebiotic era.

##### Critical View

- Nonenzymatic replication—A paper from the Szostak group [53] maintains: “it is not yet possible to copy, in an effective and prebiotically plausible manner, RNA templates long enough to encode ribozymes that might enable RNA-catalyzed self-replication processes”. Moreover, the present nonenzymatic mechanisms seem to contain fundamental pitfalls; to achieve template generality in replication, a specific primer should randomly exist in the pool for each replicated oligonucleotide, a requirement whose probability of realization is infinitesimal. Additionally, the current rate obtained for monomers addition is limited, while in the case of one pot template-directed replication via ligation, the expected competition at each elongation step [57], between the 64 possible combinatorial triplets or the 264 quartets, is bound to jam the process.
- The replicase problem—The most efficient RNA replicases generated in vitro so far had sequences of about 200 mer long. Their complexity is likely to represent an intrinsic difficulty, that is, a limited efficiency of the polymerase ribozyme caused by its weak affinity for its substrates [32,58]. Even if one assumes a much simpler ribozyme of merely 40 mer, as calculated by Joyce [59] “to represent all of these sequence combinations… would require 27 kg of RNA, which seems highly implausible”. The probability of spontaneous emergence of a complex RNA entity of about 200 mer is thus infinitesimal, even under significant relaxation of the requirement for sequence conservation.
- Cross-replication—Assuming that, despite the improbability, a single RNA replicase would have randomly emerged, the likelihood that a second ribozyme could appear at the same venue and point in time, to template-copy the first replicase, is close to none.
- Folded vs. unfolded replicases—This conundrum emanates from the fact that folded RNA ribozymes contain double-stranded regions that need to be melted in order to be copied. The requirement, in the context of the prebiotic RNA replicase, was therefore ambiguous—it had to retain its structure and function under the same environmental conditions in which the copied replicase was required to melt. To reconcile this difficulty, it was suggested that RNA replicases must have existed in a delicate balance between the folded state necessary for catalysis, and the unfolded state necessary for template activity [60]. Equilibrium, however, requires a large number of replicases, which is improbable considering the complexity of this ribozyme and more so, if it corresponds to the initial unit of evolution that should have been as simple as possible.
- Continuity problem—The transition from an RNA-only to an RNA–protein world, which must have predated LUCA, is a step function in the mathematical sense. It does not conform to the “continuity principle” that goes back to Darwin [1]. This transition was hypothesized to occur by proteins taking over catalysis [56], but such a scenario is not without difficulty, as referred to by Lanier and Williams [61]: “There is no evidence to our knowledge that Darwinian processes can revise the Molecular Toolbox or radically alter the Universal Gene Set. Available evidence suggests takeovers are unlikely by Darwinian processes”.

#### 2.2.2. RNA–DNA

An RNA–DNA unit of evolution is a set of molecules in which DNA plays the role of genetic material, encoding the RNA catalysts, while a replicase made of RNA copies the genome. According to Table 1, its sustainability is higher than that of the RNA-only unit of evolution.

##### Unit of Evolution Content

Apart from random oligonucleotides, the only obligatory component, same as in the RNA-only unit of evolution, is an efficient and accurate enough replication mode. The similarity between the chemistry of DNA and RNA allows the assumption that the initial replication mode was nonspecific, i.e., that it would copy both types of oligonucleotides. It would have transcribed DNA genes into RNA catalysts, replicated the DNA genome, and reversely transcribed the RNA sequence of spontaneously emerging ribozymes into DNA, allowing their incorporation into the genome.

##### Autonomous Advent

An RNA–DNA unit of evolution could have, in principle, resulted from the evolution of a previous RNA-only unit, implemented via template-directed copying of the RNA-genome into DNA. This transition would have been a small evolutionary step [62], being, therefore, a conceivable part of a continuous evolutionary path that would have granted the RNA-only unit with a better-performing DNA genome [43,44,45], thus with better sustainability (Table 1, set 4).

A direct path into an RNA–DNA unit of evolution can be envisioned too, in case the concurrent appearance of DNA and RNA in the prebiotic environment is assumed [63]. This would have led to the formation of heterogeneous strands consisting of RNA and DNA nucleotides, which are functional, but less efficient than pure RNA [64]. The greater stability of homogenous sequences would have resulted in the persistence of pure DNA and of pure RNA strands, while heterogeneous strands would be gradually degraded. This process was suggested to have led to a prebiological heterogeneity-to-homogeneity scenario, enabling the generation of homogenous oligonucleotides capable of efficiently fulfilling the informational and catalytic roles necessary for Darwinian evolution [65].

##### Extant Perspective

In extant biology, there are instances of RNA–DNA heterogeneity, suggesting that such heterogeneity could have been part of the evolutionary process of life [66].

##### Evolutionary Prospect

The RNA–DNA unit of evolution, similarly to the RNA-only unit, would evolve towards LUCA by incorporating functional proteins, which were suggested to take over most of the catalysis performed by RNA [56]. Only at this stage could the translation system emerge, and the resulting unit of evolution would be the most sustainable one, i.e., RNA–DNA–protein (Table 1 set 6).

##### Critical View

- Same as items 1–6 for the RNA-only unit of evolution.
- Thermodynamics—The scenario suggested for the direct formation of an RNA–DNA unit of evolution relies on a spontaneous heterogeneity-to-homogeneity process [65], which would have led from disorder to order, i.e., lowered the entropy of the system. Such a process could have taken place only at a specific temperature range that, dependent on the involved enthalpies, would have reduced the free energy of the system. The relevant temperature range was not determined, leaving the question of the feasibility of such a process unanswered.

#### 2.2.3. RNA–Protein

An RNA–protein unit of evolution is a set of molecules in which RNA plays the role of genetic material, encoding the protein catalysts, while a proto-polymerase enzyme copies RNA. It conforms to the central dogma of molecular biology, which formulates that the instructions for protein synthesis, stored in the genome and transcribed by a polymerase into mRNA, are translated on the ribosome into proteins, i.e., that contemporary translation is primarily executed by RNA and transcription by proteins. Accordingly, the RNA–protein coevolution hypothesis suggests that minimalist versions of the RNA core components of the modern translation system, together with a simple replicating enzyme, could have self-assembled in the prebiotic era into the initial unit of evolution, cross-catalyzing the formation of each other [3,26,27,28,29,30,31,32].

##### Unit of Evolution Content

An RNA–protein system is composed of two biopolymers with an intrinsic asymmetry between their replicative properties. While the sequence of any RNA string can be reproduced by two cycles of replication, peptide sequences, in general, cannot be directly copied [26] and their reproduction must rely upon preserving their sequence in an oligonucleotide [3,31]. The establishment of an injective relation that maps each amino acid to a specific combination of nucleotides (a nucleotide triplet is assumed here) is, therefore, inevitable. As a result of this asymmetry, the model of a minimal RNA–protein unit of evolution is rather complex. In addition to random RNA strings and amino acids, it is required to contain a genetic code, a processive proto-ribosome capable of translating an RNA string into a polypeptide, specifically-aminoacylated proto-tRNAs and a proto-polymerase enzyme encoded by a random RNA string. A scenario suggesting the spontaneous materialization of each of these components is offered below.

##### Autonomous Advent

An RNA–protein unit of evolution could have, in principle, originated from a former RNA world by proteins taking over most of the roles executed by the ribozymes [56]. Alternatively, it might have emerged autonomously [3,27,28,29], before any former self-sustained molecular system existed. The RNA building blocks required for assembling an RNA–protein unit of evolution, i.e., RNA strands, hairpins, and L-shaped RNAs, would have already been present in the primordial pool, being part of the chemistry era. Functional RNA components of the RNA–protein set, which are suggested to materialize via the congregation of these building blocks, i.e., proto-ribosomes and proto-tRNAs, are requested to meet three prerequisites: a feasible likelihood to emerge spontaneously, sustainability, and the capacity to continuously evolve into their modern descendent.

##### Advent of the Proto-Ribosome

The autonomous emergence of a proto-ribosome would have probably occurred in two stages:

- Autonomous materialization of a simple noncoded proto-ribosome that could catalyze peptide bond formation between two random amino acids, i.e., proto-LSU.
- Autonomous advent of a minimal coded proto-ribosome that was capable of processively translating a code written in an RNA string into a code-directed polypeptide.

**Figure 1:**
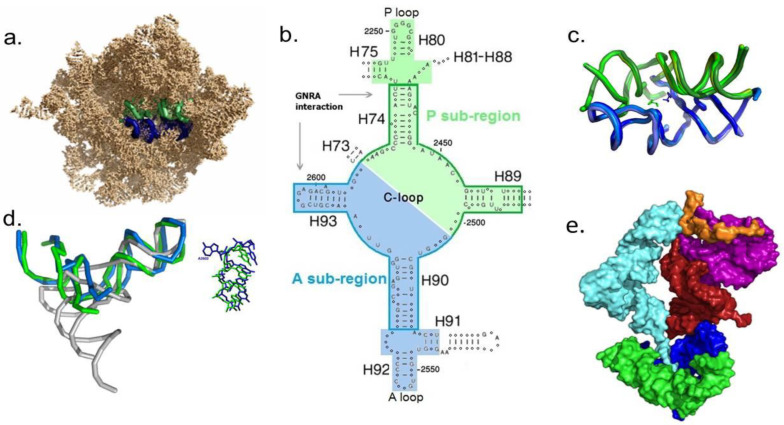
The proto-ribosome: (**a**) The symmetrical region within the 23S rRNA of *E. coli* ribosome (PDB code 2AW4). The A- and P-subregions, in blue and green, respectively, throughout. Helices H68-H71 were removed to reveal the PTC area. (**b**) 2D scheme of the symmetrical region (on colored background), drawn in a manner exhibiting the twofold symmetry, with the central loop of domain V (C-loop) at its center. The sequence assigned to the remnant of the DPR (boundary marked) is composed of two L-shaped molecules, the A- and P-DPR monomers. Nucleotides conserved by more than 97% in each of the three life domains, as detected in the CRW site [77], are presented by capital letters and the remaining nucleotides by circles. (**c**) Overlap of the DPR fold as found in the high-resolution structures of archaea (PDB code 1VQ6), bacteria (PDB code 2WDL), and eukarya (PDB code 3U5D) ribosomes, portraying its extreme tertiary conservation in the three life domains. The pocket is projected approximately along the symmetry axis, with the reactants (PDB code 2WDL) positioned at the bottom of the cavity. (**d**) Overlap of the A- and P-DPR monomers from *E. coli* (PDB code 2AW4), obtained by rotating one monomer by 179.6° around the symmetry axis. The projection direction is perpendicular to the one shown in (**c**). tRNA molecule (PDB code 4TRA, in gray) is overlaid with its anticodon arm overlapping H89 and H93 helices from the P- and A-monomers, respectively. Magnified nucleotides from the superimposed stems of H89 and its symmetry-related H93 depict the conformational match. Nucleotide A2602, which is functionally active, bulges into the PTC and breaks the overall symmetry. (**e**) Model of a minimal coded proto-ribosome assembled from four L-shaped entities of about 60–70 nucleotides each (derived from PDB code 1VY4), i.e., the A-, P-DPR monomers, the proto-SSU (purple), and the bridging element (dark red), complexed with mRNA (orange) and tRNA (cyan).

Advent of the noncoded proto-ribosome—The region encircling the PTC is highly conserved [67,68], both structurally and sequentially (Figure 1a–c), in ribosomes from all life domains. This region is widely held to be the oldest part of the large ribosomal subunit, being the relic of an initial noncoded proto-ribosome that catalyzed peptide bond formation between two random amino acids [29,69,70,71,72,73,74,75]. Within the contemporary ribosome, which generally lacks any internal symmetry, this region has a pocket-like shape possessing an approximate twofold rotational symmetry, which relates the 3D folds and the nucleotide conformation of its two halves (Figure 1c,d), but not their sequences [68]. The amino acid reactants are symmetrically accommodated in the PTC [76], in a stereochemistry optimal for peptide bond formation (Figure 1c). Based on the symmetry, a model of a standalone, noncoded proto-ribosome termed the Dimeric Proto Ribosome (DPR) was derived from the modern LSU [29,73], where it forms a dimer of two L-shaped RNA molecules of about 60 nucleotides each, which are symmetrically associated around a twofold rotation axis (Figure 1b–d). The size and layout of the two monomers are comparable to that of tRNA (Figure 1d).

The ability of the DPR to spontaneously emerge from random oligonucleotides of sufficient length relies on two properties: (a) the energetic downhill processes of its folding and dimerization; and (b) the limited sequence specificity required for preserving its structure and function:

(a) Energetic considerations for the DPR formation—Free energy minimization was performed with Mfold [78] on the DPR-monomer sequences derived from several contemporary bacterial and archaeal ribosomes. The results demonstrated that if any of these sequences existed in the prebiotic world, it would be compelled to fold into an L-shaped element matching the one found within the modern ribosome [29]. Moreover, their dimerization, via the GNRA interaction (Figure 1b), has a stabilizing effect [79], promoting the spontaneous assembly of DPR pockets.

(b) Statistical considerations for the DPR formation—The feasibility of the accidental occurrence of an RNA segment that can preserve both the structure and the function of the DPR monomer was statistically analyzed [80]. The identity of nucleotides essential for maintaining the function, i.e., those highly conserved in all life domains, as well as that of nucleotides which secure the secondary and tertiary structures of the PTC region, was retained, while the identity requirements for the remaining nucleotides were relaxed. The analysis indicated that each liter of 1 mM solution of random RNA chains of 60 mer would have included about 300 oligonucleotides having sequences predisposed to form the L-shaped monomers of the DPR. These monomers would possess dimerization affinity and conserve the reactants accommodation sites, suggesting that DPR pockets that could catalyze peptide bond formation were reasonably common. Furthermore, replication of the DPR monomers would have been relatively simple and efficient. Sequence complementarity between the C-loop nucleotides that form the PTC cavity in the A- and P-monomers of the DPR (Figure 1b), which was observed in bacterial ribosomes [81], suggests that the strand of each monomer could have acted as template for the synthesis of its complement, forming a self-replicating ribozyme which had an enhanced capacity to proliferate.

And, indeed, in agreement with the hypothesis [29,73], most of the RNA constructs of DPR monomers derived from sequences of the PTC region from several bacteria were recently reported to fold and dimerize spontaneously in vitro [82]. More importantly, the dimers were found to mediate peptide bond formation and to synthesize up to 9 mer peptides [82,83]. Taken together, these results indicate that the DPR, i.e., the proto-LSU, would have had an acceptable probability to self-assemble in the prebiotic era from random RNA chains. The double-stranded arms of the L-shaped monomers, together with the stabilization enhanced by dimerization, guaranteed the sustainability of the pockets, while being derived from the modern ribosome ensures its capacity to continuously evolve towards the contemporary LSU. The DPR thus meets all three prerequisites of autonomous emergence, sustainability, and continuous evolution, while its ability to emerge spontaneously is experimentally supported [82,83]. Such pockets could have therefore been floating in the primordial pool, accommodating random amino acids, and providing the positional catalysis required for forming di-peptides and short polypeptides of random composition. The synthesized peptides would be distinguished from the mineral-catalyzed peptides by being homochiral, due to the preference of the PTC, and its derived DPR, for L-amino acids [84]. Such random peptides, especially when positively charged, could have, in turn, stabilized the proto-ribosomes.

Advent of a coded proto-ribosome—A minimal proto-ribosome that was capable of processively translating a code written in a random RNA string into a code-directed polypeptide would have to be composed of the following components: (a) a noncoded proto-LSU, where peptide bond formation took place; (b) a minimal RNA entity containing the active site of the SSU, where the primordial mRNA was accommodated and the specifically aminoacylated proto-tRNAs were recognized via their anticodon (AC) loop; and (c) a bridging RNA element that filled the gap between the two active sites, adjusting the distance between them to the size of the proto-tRNA.

A hypothetical model derived from the structure of the contemporary 70S ribosome suggests that the merger of the two L-shaped DPR monomers with two additional L-shaped RNA elements, i.e., with the proto-SSU which was composed of SSU helices h44, h45 (nucleotides 1400–1419; 1481–1531), and the bridging element comprised of H69–H71 from domain IV of the LSU (nucleotides 1906–1968), could have assembled a complete coded proto-ribosome (Figure 1e) [85]. The thermodynamically favorable folds of the proto-SSU and of the bridging element, each consisting of about 60–70 nucleotides, were predicted by performing free energy minimization with Mfold [78], using the corresponding *E. coli* and Thermus thermophilus sequences. Both regions acquired folding schemes analogous to those found within the contemporary ribosome, with a negative change in their free energy, indicating their self-foldability and stability. Thus, these two RNA elements, lasting in the prebiotic pool, could have been added over time to the noncoded DPR, through specific A-minor interactions which are still observed in the modern ribosome, yielding a minimal coded proto-ribosome (Figure 1e) that encompasses only 6% of the current 70S rRNA sequence [85].

##### Advent of the Proto-tRNA

**Figure 2:**
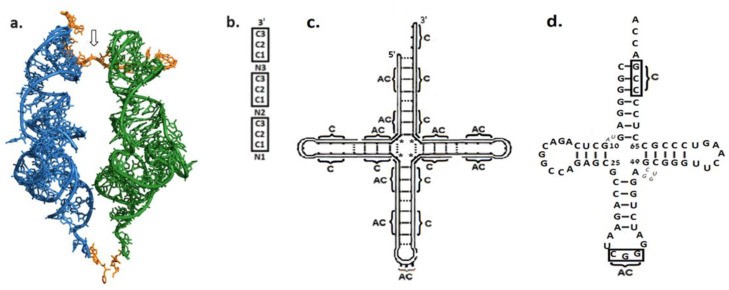
(**a**) A-, P-site tRNAs in the structure of Thermus thermophilus 70S ribosome complexed with mRNA and tRNAs (PDB code 1VY4), forming a rhombus-like arrangement that allows them to simultaneously bind to neighboring codons on mRNA (in orange) and to bring the amino acid reactants (in gold) to within the required proximity. The kink in mRNA is marked by an arrow. (**b**) A 12 mer RNA strand that carries three copies of the codon C1C2C3 (in thick frame) together with three nonspecific nucleotides (N) separating them can act as a building block for the self-assembled proto-tRNA. (**c**) Formation of a cloverleaf scheme, through the assembly of 3 copies of the strand in (**b**) and 3 copies of its complement. Solid lines represent Watson–Crick base pairs and dashed lines—potential base pairs occurring when nonspecific nucleotides accidentally complement. Non-specific nucleotides required to base pair for assembling the 4-arm scheme are depicted by asterisks. Disconnected points on the outer line symbolize points of ligation of the 12 mer strands. (**d**) Secondary structure of tRNA^Pro^ from Thermotoga maritima [90] showing high compatibility with the assembly model (**c**). The cognate coding triplet CCG, found in positions 70–72 in 98% of the acceptor stems from bacterial tRNA^Pro^ and the corresponding AC in the anticodon loop, are marked. Pseudouridine in the T-stem is referred to as U. Nucleotides lacking counterparts in the assembly model (**c**) are indicated by smaller italic letters.

The modern translation uses L-shaped tRNAs as mediators between the mRNA codon, accommodated on the SSU, and its corresponding amino acid, placed in the PTC. Charged hairpins were proposed to have preceded the tRNAs, playing the mediator role in early translation. By lining up, side by side, along an RNA string serving as mRNA, hairpins were suggested to enable the formation of a coded peptide [86,87,88]. However, the subsequent determination of high-resolution structures of 70S ribosomes complexed with tRNAs bases paired to neighboring codons on an mRNA string, clearly demonstrated that such hairpins are bound to collide. The maximal length measured for a single codon triplet is 18.2 Å, while the width of the AC stem ranges between 20.6–21.7 Å [85]. In the modern ribosome, the stems collision is prevented by a kink in mRNA, generated via its interaction with SSU helix 44 [89]. The kink separates the AC stems of the two tRNAs, and only the perpendicular acceptor-TΨC arms, which are inclined towards each other, can place the reacting amino acids in the PTC (Figure 2a). Hairpins, therefore, would have failed to simultaneously connect their AC loops to neighboring codons, while bringing the reacting amino acids to the required proximity [85]. These steric considerations, combined with the conformity to the continuity principle [1], indicate that the proto-tRNA molecules which took part in the primordial translation were already L-shaped. However, it cannot be ruled out that the initial L-shaped tRNA had shorter stems, and, correspondingly, that a smaller bridging element mediated the interaction between the proto-LSU and proto-SSU.

Two central views concerning the prebiotic assembly of an L-shaped tRNA were previously presented. The first, which is derived from the tertiary L-shape of the modern tRNA, divides the molecule into an older part, the coaxial acceptor-TΨC half, which was suggested to be the initial proto-tRNA, and to the later-appearing anticodon-DHU half [91,92]. The second approach assumes that the prebiotic tRNA emerged via the conjugation of two hairpins, analogous to the 3′ and 5′ halves of the modern cloverleaf scheme, having either complementary sequences or nearly identical sequences obtained via duplication [93,94,95,96,97]. Both approaches, however, carry inherent difficulties. The first proto-tRNA lacks the second arm, which is indispensable for participating in translation (Figure 2a) [85], while the second approach requires the accidental occurrence of two sequence-related RNA strands of about 40 mer, being either complementary or nearly identical, which, in the absence of a replicase, seems highly implausible.

A different approach was proposed recently [98]. An RNA entity highly compatible with the modern tRNA was obtained from six short RNA strings: three copies of an initial 12 mer oligonucleotide carrying an array of three coding triplets (Figure 2b) and three copies of its complement. Such an initial RNA strand can be found in more than 0.02% of the 12 mer oligonucleotides with random composition, i.e., about 1 in 5000 random 12 mer oligonucleotides could have served as a building block of this cloverleaf proto-tRNA. Spontaneous replication of the initial 12 mer RNA strand, unzipping the duplex under change in environmental conditions, and recombination via shifted base pairing to form the cloverleaf model, are feasible chemical reactions in a prebiotic environment lacking biological catalysts. The assembly of the six strings results in a model (Figure 2c) that preserves the size, the primary and the secondary constraints that underlie the three-dimensional folding of the modern tRNA structure (Figure 2c,d). It naturally yields a 3′ end tail of four nucleotides, allows tertiary base pairing between the D-T loops which secure the L-shape, and guarantees the presence of coding triplets at the beginning of the acceptor stem and in the anticodon loop.

All in all, conditional on the existence of large amount of oligonucleotides of up to 100 mer in a prebiotic site [17,18], each of the RNA components of the RNA–protein unit of evolution, i.e., the proto-ribosome, a random RNA strand acting as mRNA, and L-shaped proto-tRNAs, would have had a reasonable likelihood to materialize autonomously in the prebiotic pool, to exhibit a rather long life span and to evolve into its modern descendant.

##### Aminoacylation of Proto-tRNAs and the Origin of the Genetic Code

The intrinsic asymmetry between the replicative properties of an RNA string, which can be reproduced by two cycles of replication, and a peptide sequence, which cannot be directly copied [26], necessitates the establishment of a code that can preserve the peptide sequence in an RNA string [3,31]. Several hypotheses concerning the emergence of the current genetic code were proposed, as reviewed by Koonin [99]. The stereochemical hypothesis suggested by Carl Woese [100], which assumes chemical attraction between certain amino acids and their coding triplets, accords best with the scenario proposed here for the autonomous emergence of an RNA–protein unit of evolution.

To execute its role in translation, the L-shaped proto-tRNA had to carry an anticodon triplet in the AC loop and to be charged with its cognate amino acid at the 3′ end. In modern biology, aminoacylation of tRNAs is carried out by synthetases, specific enzymes that aminoacylate each of the 20 tRNAs with their cognate amino acid. However, such a prebiotic mode of action would pose a chicken and egg conundrum; charging the proto-tRNA required a synthetase, which demanded aminoacylated proto-tRNAs for its synthesis. A mechanism that bypasses this conundrum, i.e., self-aminoacylation of proto-tRNAs [98], could have been applicable when a pair of cognate codon and AC triplets occupied the first three base pairs of the acceptor stem (positions 1–3:70–72), in addition to the anticodon triplet present in the AC loop. In that case, in accordance with Carl Woese’s stereochemical hypothesis [100], the distinct electrostatic landscape of the codon:AC nucleotides would form a “nest”, that will promote accommodation of the cognate amino acid via the stereochemical affinity. The proximity of the amino acid to the 3′ end would facilitate a nonenzymatic esterification occurring via folding back of the adjacent 3′ end tail, analogous to the process shown to take place in a small ribozyme having a 3′ tail of four nucleotides [101]. Self-aminoacylation of tRNA^Phe^, which was reported to occur under high pressure in the absence of ATP and synthetases [102], opens the possibility that the prebiotic self-aminoacylation may have taken place under high pressure as well, such as would prevail in submarine hydrothermal vents that were associated with the origin of life on Earth [103].

In support of this self-aminoacylation mechanism, analysis of large sequence datasets acquired from the tRNAdb data base [90] revealed extreme conservation of cognate coding triplets in specific positions on the Ala, Gly, His, Pro, and Ser bacterial acceptor stems [104]. The proto-tRNAs of these amino acids, which are mostly held to be early-emerging [99], could have, therefore, participated in such a primordial self-aminoacylation process, whereby an amino acid inhabited on its cognate stem coding triplets was nonenzymatically esterified to the 3′ end, yielding a correctly charged proto-tRNAs. These ancestral coding triplets could have later established the recognition scheme between the proto-tRNAs and the primordial synthetases, when emerged, and their retention in distinct modern tRNAs was attributed to their role as recognition elements in the aminoacylation mode utilized by class IIa synthetases [104]. These conserved coding triplets, which are assumed to have controlled the specific accommodation of amino acids on their cognate proto-tRNAs, are therefore proposed to underlie the initial genetic code [98,105] that was later supplemented, resulting in the current codon set.

Once aminoacylated proto-tRNAs were added to the other self-assembled RNA components, they could have cooperated, translating arbitrary RNA chains into random polypeptides via a factor-free protein synthesis mechanism, possibly using the nonenzymatic translocation which was reported to occur when translation was carried out by a ribosome lacking the SSU protein s12 [106]. s12 was suggested to block the spontaneous translocation in modern ribosomes, implying that its absence in the suggested coded proto-ribosome, which was composed solely of RNA, may have allowed spontaneous translocation, resulting in the synthesis of the first code-directed polypeptides.

##### Advent of the Proto-Polymerase

A portion of the random polypeptides synthesized by the proto-ribosome, which exceeded 30 residues in length, being therefore sufficiently long to allow stable folding, could have possessed limited catalytic abilities of some sort [33,34,35]. A single event where the translation of a random RNA strand yielded a protein with weak polymerase activity, possibly resembling the double-psi β-barrel (DPBB) domain in RNA polymerases [32,107], would have added a proto-polymerase to the contemporaneous RNA components and completed the RNA–protein unit of evolution. This proto-polymerase should have been at least 30 residues long, meaning that the length of the random RNA chain encoding it would be over 100 mer, making it highly improbable to be accidentally found in the prebiotic pool. However, two aspects may significantly increase the probability of its occurrence. First, the likelihood of its transpiring is inversely proportional to the number of amino acids involved in translation. Thus, restricting the number of amino acids that participated in early translation, as was previously suggested ([99] and refs therein), would have increased the likelihood of the proto-polymerase advent. Secondly, unlike the ribosome whose core is conserved in all life domains, indicating a single ancestor, the sequence and structure of modern polymerases reveal that they belong to at least five evolutionarily unrelated folds [107]. All these enzymes use a similar basic biochemistry, but variability of their sequence and structure is likely to indicate that enzymes that replicate oligonucleotides have tolerance to significant sequence mutability. Once emerged, this proto-polymerase would have copied the autonomously materialized RNA components of the RNA–protein unit of evolution. The RNA components, in turn, would have recurringly translated the initial strand coding for the proto-polymerase, thus guarantying a constant presence of a replicating enzyme in the unit of evolution, yielding an Auto Catalytic Set (ACS) [108] that signified the transition into Life [25,26,29,31,69]. 

##### Evolutionary Prospect

This RNA–protein system would have initially evolved by adding new enzymes acquired via the translation of arbitrary RNA chains. Alongside, the A- and P-loops would be added to the DPR through expansion from the tips of helices H90 and H74, respectively (Figure 1b). This would have allowed base pairing of the tRNAs 3′ end to H92 and H80 loops, stabilizing the proto-tRNAs residing in the A-, P-sites, thus assisting peptide bond formation. The structure of this extended proto-LSU essentially overlaps the cumulative product of phases 1 and 2 in the accretion model presented by Williams and coworkers [75]. Subsequently, the rRNA of the coded proto-ribosome would expand over time, by incorporating random RNA elements, possibly via A-minor interaction [72], helix elongation, and addition of expansion segments [75], further enhancing the stabilization of the accommodated proto-tRNAs. Sequence variability, resulting from the limited replication accuracy expected from the initial proto-polymerases, would have enabled Darwinian selection of the fittest RNA components within each unit of evolution.

The incidental rise of a new functional protein would have granted its unit of evolution with a selective advantage. One of the earliest enzymes to emerge would have probably been a nonspecific synthetase [2,31]. By accommodating an ATP molecule and adhering to an acceptor stem of a proto-tRNA carrying a cognate amino acid accommodated via the stereochemical affinity, the enzyme could have activated the amino acid and promoted its esterification to the nearby 3′ end tail. In the next evolutionary stage, a specific synthetase of limited size, which was suggested to include merely the modern catalytic domain, would have emerged. It would accommodate the cognate amino acid and an ATP molecule, catalyze amino acid activation, and achieve specific recognition of its cognate proto-tRNA only via the coding triplets contained in the tRNA acceptor stem [91,109]. The existence of such conserved coding triplets only in bacterial tRNAs charged by the modern class IIa synthetases suggests that their ancestor is likely to have been the first specific synthetase to emerge [104,110]. Later, when the prebiotic synthetase acquired a moiety that could interact with the anticodon loop, the AC triplet became the primary identity determinant in most aminoacylation processes [111].

Interestingly, the active sites of the two key enzymes in the RNA–protein unit of evolution, the polymerase and the synthetase, consist of β strands, in accordance with the suggestion that the active sites of early enzymes consisted of β-sheets and β-turns [2,112,113]. Further coevolution of the primitive translation system, together with the initial proteins, could have outlined a continuous path from the RNA–protein unit of evolution into LUCA.

##### Extant Perspective

The RNA–protein unit of evolution contains the essence of contemporary replication and translation processes, which stands at the heart of modern life. Its prebiotic existence, prior to LUCA, either resulting from the evolution of a former RNA world or from its direct materialization, seems therefore uncontestable.

##### Critical View

- Complexity—Although each of the RNA and protein components composing the RNA–protein unit of evolution seems to have an acceptable probability to materialize spontaneously, the whole process is extremely complex compared with the simple emergence of an RNA world, and the likelihood of its occurrence is significantly lower.
- Experimental verification—The capability of a noncoded dimeric proto-ribosome to assemble spontaneously and catalyze the synthesis of peptides was already shown experimentally [82,83]. The self-assembly of L-shaped proto-tRNAs from 12 mer RNA strands, as well as their self-aminoacylation, are experimentally testable. However, the possibility of lending experimental support to the self-assembly of the minimal coded proto-ribosome model [85], through the merger of the DPR with the L-shaped proto-SSU and the bridging element (Figure 1e), seems questionable. The proto-SSU sequence is less conserved than the other components of the model, while the bridging element contains a single-stranded segment of 20 nucleotides (LSU nucleotides 1925–1944), which may form different globular structures under varying conditions. The probability of obtaining each of these two moieties in the laboratory in their ancestral form is, therefore, minute, and an attempt to combine them with the DPR to yield a peptide-forming molecular machine seems extremely challenging.
- Stereochemical affinity?—According to the present scenario, the emergence of the genetic code and of the proto-tRNA self-aminoacylation are conditional on the verity of Woese’s stereochemical theory [100]. Up till now, this hypothesis has been tested mainly on aptamers [114] and within the ribosome [115], giving inconclusive results [99]. Initial docking simulations seem to indicate an enhanced preference of certain early-appearing amino acids towards their cognate coding triplet located in the tRNA acceptor stem (unpublished results), but credibility requires experimental support.

#### 2.2.4. DNA–Protein

The DNA–protein unit of evolution is a set of molecules in which enzymes perform all the catalytic tasks, while DNA plays the role of genetic material, encoding the protein catalysts.

##### Unit of Evolution Content

The minimal version of such a set would have included a DNA genome, a genetic code, a proto-polymerase enzyme that copied the genome, and a proteinaceous ribosome-analog that translated the encoding genes into enzymes.

##### Autonomous Advent

This molecular set could have, in principle, evolved from a preceding RNA–protein world, through template-directed copying of the RNA genes into DNA. The transition would have granted the unit of evolution with a better-performing genome but would cause the loss of the RNA catalytic abilities, requiring the exchange of a proto-ribosome made of RNA with a ribosome-analog which is a protein. The ribosome-analog enzyme could have emerged autonomously solely via a completely improbable synchronization of two low-chance events; an arbitrary polypeptide should fold to form a protein with some translational abilities, and this transient protein is required to translate a random DNA string that miraculously exists at the same time and place, encoding the sequence of the constant ribosome-analog enzyme of the set.

##### Extant Perspective

Many viruses are DNA–proteins systems, but, as emanates from the aforesaid analysis, the lack of ribosomes impedes their autonomous existence, compelling them to rely upon the translation systems of living organisms.

## 3. Discussion

Four different sets of biopolymers were examined for their suitability to form the initial unit of evolution, which could have launched the transition from the inanimate world towards life as we know it. One model, the DNA–protein unit of evolution, was immediately rejected, due to the requirement to contain a ribosome-analog enzyme, which could not have autonomously materialized in the unit of evolution.

A second set, the RNA-only, is the equivalent of the RNA world hypothesis, an attractive idea in which self-folding of random RNA oligonucleotides could have resulted in a unit of evolution having both functionality and heredity. However, a feasible mode of replication that would guarantee the continuity of this unit of evolution has not been figured out yet. The existence of a replicase made of RNA that could copy a general RNA sequence seems essentially impossible; it would be inherently complex due to its weak affinity for its substrates [32,58], and the concurrent formation of two of these ribozymes from random RNA chains, such that one could copy the other, is highly improbable. Moreover, a physical environment where one polymerase made of RNA would have retained its structure and function while copying the second unfolded one seems inconceivable.

A better feasibility to serve as the prebiotic replication mechanism in an RNA-only unit of evolution is offered by the nonenzymatic replication. The wide-scale experimental efforts applied so far ([53] and refs therein) involved primer extension, i.e., they require, for each copied string, a primer with a distinct complementary sequence whose arbitrary existence in the prebiotic surrounding seems highly implausible. Nor is the template-directed replication via ligation plausible, due to the expected competition between the 64 nucleotide triplets or 256 nucleotide quartets, which would have jammed the elongation. Achieving higher confidence in the prebiotic existence of an RNA-only unit of evolution, that is, of an RNA world, is therefore conditional on the ability to present a proper replication mode that could have nonenzymatically copied longish RNA chains with adequate accuracy. All the reservations pertinent to the RNA-only unit, and more, are applicable to the RNA–DNA unit of evolution.

In contrast with the simplicity of the RNA world hypothesis, the formation of the fourth set, the RNA–protein unit of evolution, is complex. It is a multistep process, where the spontaneous occurrence of each step seems energetically feasible, but its likelihood is minute. To cope with the probability issue, Koonin suggested estimating the likelihood of an analogous scenario under the assumption that it could have occurred anywhere in the universe and not specifically on Earth [28]. This inflation, however, is not necessarily needed. The materialization of an RNA–protein unit of evolution on Earth may still be possible because, as far as we know, life emerged here only once, pointing to initiation via an extremely rare event. The low, but realistic, probability that coded proto-ribosomes and aminoacylated proto-tRNAs could have materialized again and again in the prebiotic pool over time, followed by the one-time advent of a primitive polymerase through the translation of a random RNA chain, might have been the rare event that marked the starting point of evolution towards life as we know it.

A weak point In in the RNA–protein scenario presented here is its reliance on the existence of a stereochemical affinity between certain amino acids and their coding triplets [100], an attraction whose verification efforts gave, so far, nonconclusive results [99]. Initial docking simulations did seem to indicate an enhanced preference of certain early-appearing amino acids towards their cognate coding triplet located in the tRNA acceptor stem (unpublished results), but the centrality of Woese’s hypothesis in origin of life scenarios calls for experimental examination. Testing the accommodation of Ala, His, Gly, Pro, and Ser on their coding triplets contained in the acceptor stems of their cognate tRNAs [104], in accordance with the self-aminoacylation mechanism [98], may help substantiate or refute the verity of the stereochemical affinity. Another intriguing line of experimental research would involve testing the existence of this affinity under high pressure, following the self-aminoacylation of tRNA^Phe^, reported to occur under high pressure in the absence of ATP and synthetase [102].

Despite these difficulties, a scenario suggesting the emergence of life in an RNA–protein world seems to offer significant advantages over the RNA world hypothesis:

- An initial RNA–protein system could have continuously evolved into the current RNA–DNA–protein world, without the need to go through a discontinuous step of transferring most of the catalysis from RNA to proteins, as the RNA world hypothesis entails.
- The chemical versatility and efficiency of enzymes would have been beneficial in promoting the emergence of life.
- The self-aminoacylation mechanism [98] discussed here requires only a “soft“ version of Woese’s stereochemical affinity. It suffices that a certain amino acid would have higher affinity towards its cognate coding triplets, compared to that exhibited by the limited number of the other contemporaneous amino acids, to allow the formation of conceivable percentage of correctly aminoacylated tRNAs; thus, of correctly translated proteins.
- The occurrence of the statistically-challenging step in the RNA–protein scenario, i.e., the advent of a proto-polymerase via the prebiotic translation of a random RNA chain that accidentally encoded it, seems to be inevitable in any scenario. Being the only pathway whereby the proto-polymerase could have established a constant presence in a primordial unit of evolution prior to LUCA implies that this step would have occurred whether the route to LUCA went through an RNA world or directly into an RNA–protein world.
- The scenario presented here for the autonomous advent of the protein-RNA unit of evolution is, in principle, experimentally verifiable. The capability of a noncoded dimeric proto-ribosome to assemble spontaneously and catalyze the synthesis of peptides was already demonstrated experimentally [82,83], while the self-assembly of L-shaped proto-tRNAs from 12 mer RNA strands, as well as their self-aminoacylation, are testable [98]. Obtaining a coded proto-ribosome and an enzyme possessing some replicative abilities should require wide-scale in vitro directed evolution experiments and a great deal of luck, but the attempt is doable. In contrast, the means for verifying “proteins taking over catalysis by RNA” [56], as implied by the RNA world hypothesis, are not present [61].
- The present scenario for the advent of an RNA–protein unit of evolution bypasses the “chicken and egg” conundrum posed by an enzymatic prebiotic aminoacylation, which requires that proto-tRNAs would be specifically aminoacylated by synthetases that needed specifically aminoacylated tRNAs for their formation. Autonomously formed and self-aminoacylated proto-tRNAs, being part of the RNA–protein unit of evolution, could have participated in the synthesis of the initial synthetase by translating a random RNA string encoding it.
- A situation where the two central types of polymerases in the living cell, i.e., the ribosome that polymerizes amino acids and the polymerase that polymerizes nucleotides, are formed from different polymers with distinct environmental sensitivities is advantageous. Under stress conditions that specifically affect proteins, such as a denaturating agent that melts proteins but not RNA, a ribosome could have recovered the content of the unit of evolution by producing additional proteins, while a ribosome-analog enzyme would be disabled. Symmetrically, under RNA stress conditions, the enzyme polymerase may still function to regenerate the corrupted RNA components [29]. This notion seems to provide a proper answer to a long-standing question, first posed by Crick [21], concerned with the preference of nature to adhere to a ribosome which is a ribozyme, rather than transferring the translation process to a more efficient protein.

A favorable surrounding that could have promoted the advent of an RNA–protein world is offered by the submarine hydrothermal vents [103]. In that environment, amino acids could form via serpentinization [12], liquid under thermal gradient can yield long oligonucleotides [17], elevated pressure may assist self-aminoacylation [102], abiotic compartments in the form of pores in the chimney’s rocks should be common, constant supply of essential minerals and ions is assured, and thermal energy is abundant, while oscillation in the environmental conditions could promote nucleotide formation [116] and nonenzymatic replication [55,117].

Analysis of biopolymer combinations that may have participated in the autonomous emergence of life seems to lend considerable support to the direct rising of an RNA–protein unit of evolution, and the recent report of an in vitro synthesis of peptides by a standalone dimeric analog of the LSU core [82,83] further corroborates it. This experimental result gives hope that additional simplified life processes, suggested by the RNA–protein model, would be observed in a test tube, confirming that this model offers a plausible starting point for a pathway whereby the inanimate material could have naturally evolved into the complex protein biosynthesis system, shared by all the extant living organisms.

## Figures and Tables

**Table 1:** Ranking of biopolymer sets. Zero, one, and two asterisks indicate weak, moderate, and high performance of the polymer in a specific role, respectively. In parenthesis—the relevant polymer.

Hypothetical Set	Polymers Content of the “Unit of Evolution”	Score as Genetic Material	Score as Catalyst	Rank = Genetic × Catalytic Scores
1	Protein	0 * (Protein)	2 * (Protein)	0 *
2	DNA	2 * (DNA)	0 * (DNA)	0 *
3	RNA	1 * (RNA)	1 * (RNA)	1 *
4	RNA–DNA	2 * (DNA)	1 * (RNA)	2 *
5	RNA–protein	1 * (RNA)	2 * (Protein) + 1 * (RNA) = 3 *	3 *
6	DNA–protein	2 * (DNA)	2 * (Protein)	4 *
7	RNA–DNA–protein	2 * (DNA)	2 * (Protein) + 1 * (RNA) = 3 *	6 *

## Data Availability

Data sharing not applicable.
